# Association between allostatic load and adverse outcomes among older patients with heart failure with preserved ejection fraction

**DOI:** 10.1186/s12877-023-04091-x

**Published:** 2023-06-09

**Authors:** Benchuan Hao, Jianqiao Chen, Yulun Cai, Huiying Li, Zifan Zhu, Weihao Xu, Hongbin Liu

**Affiliations:** 1 Department of Cardiology, The Second Medical Centre, Chinese PLA General Hospital, Beijing, China; 2 Medical School of Chinese PLA, Beijing, China; 3 Beijing Key Laboratory of Chronic Heart Failure Precision Medicine, Beijing, China; 4 Department of Cardiology, Guangdong Provincial People’s Hospital, Guangdong Provincial Cardiovascular Institute, Guangdong Academy of Medical Sciences, Guangzhou, China; 5 Department of Geriatrics, Guangdong Provincial Geriatrics Institute, Guangdong Provincial People’s Hospital, Guangdong Academy of Medical Sciences, Guangzhou, China; 6 Department of Geriatrics, Henan Provincial People’s Hospital, Zhengzhou, China

**Keywords:** Allostatic load, Heart failure with preserved ejection fraction, Older people, Mortality, Hospital admission.

## Abstract

**Background:**

The allostatic load (AL) refers to the cumulative weakening of multiple physiological systems caused by repeated adaptation of the body to stressors There are still no studies have focused on the association between AL and the prognosis of patients with heart failure with preserved ejection fraction (HFpEF). The present study aimed to investigate the association between AL and adverse outcomes, including mortality and HF admission, among elderly male patients with HFpEF.

**Methods:**

We conducted a prospective cohort study of 1111 elderly male patients with HFpEF, diagnosed between 2015 and 2019 and followed up through 2021. We constructed an AL measure using a combination of 12 biomarkers. The diagnosis of HFpEF was made according to the 2021 European Society of Cardiology guidelines. A Cox proportional hazards model was used to determine the associations between AL and adverse outcomes.

**Results:**

In multivariate analysis, AL was significantly associated with increased risk of all-cause mortality (medium AL: adjusted hazard ratio [HR] = 2.53; 95% confidence interval [CI] 1.37–4.68; high AL: HR = 4.21; 95% CI 2.27–7.83; per-score increase: HR = 1.31; 95% CI 1.18–1.46), cardiovascular mortality (medium AL: HR = 2.67; 95% CI 1.07–6.68; high AL: HR = 3.13; 95% CI 1.23–7.97; per-score increase: HR = 1.20; 95% CI 1.03–1.40), non-cardiovascular mortality (medium AL: HR = 2.45; 95% CI 1.06–5.63; high AL: HR = 5.81; 95% CI 2.55–10.28; per-score increase: HR = 1.46; 95% CI 1.26–1.69), and HF admission (medium AL: HR = 2.68; 95% CI 1.43–5.01; high AL: HR = 3.24; 95% CI 1.69–6.23; per-score increase: HR = 1.24; 95% CI 1.11–1.39). Consistent results were found in multiple subgroup analyses.

**Conclusions:**

A higher AL was associated with poor prognosis in elderly men with HFpEF. AL relies on information that is easily obtained in physical examinations and laboratory parameters and can be assessed in various care and clinical settings to help risk stratification of HFpEF patients.

**Supplementary Information:**

The online version contains supplementary material available at 10.1186/s12877-023-04091-x.

## Background

Heart failure with preserved ejection fraction (HFpEF) is a highly heterogeneous clinical syndrome with high prevalence, which accounts for approximately half of all patients with heart failure (HF) and continues to increase at an astonishing rate of up to 1% per year [1, 2]. HFpEF has a poor prognosis, with high mortality and hospital admission rates similar to HF with reduced ejection fraction [2]. Currently, HFpEF is believed to be associated with aging, comorbidities, and multiple organ dysfunction [3–5], and their complex interrelationship reduces the prognostic ability of individual biomarkers for HFpEF. Therefore, a multi-factor assessment system is needed to measure the dissonance between different physiological systems in patients with HFpEF [6].

The theory of allostatic load (AL) was first developed by McEwen and colleagues in 1993 and is derived from the definition of “allostatic”. AL refers to the cumulative weakening of multiple physiological systems caused by repeated adaptation of the body to stressors [7]. This includes hormonal activation during stressful events (primary mediators) and system-level physiological responses caused by fluctuations in primary mediators (secondary outcomes), such as changes in blood pressure and metabolic disorders. The breakdown of multi-system regulation caused by repeated adaptation ultimately leads to the occurrence of disease [8]. The determination of AL lacks a gold standard and is often constructed using a combination of multi-system biomarkers that reflect primary mediators and/or secondary outcomes [9, 10].

The AL reflects physiological dysregulation across several biological systems and has been shown to predict the risk of certain major physical and mental health outcomes [11–15]. Mattei et al. [13] reported that a higher burden of AL among older people in Puerto Rico was significantly associated with increased rates of abdominal obesity, hypertension, diabetes, cardiovascular disease, and arthritis. Studies have shown that [16] a high AL burden is associated with all-cause and cardiovascular mortality among adults in the United States. Additionally, AL has been shown to mediate the occurrence of coronary heart disease in association with educational level or depression [17, 18]. However, no studies have focused on the association between AL and the prognosis of patients with HFpEF. Therefore, in this study, we investigated the association between AL and mortality and HF admission rates in older male patients with HFpEF. We hypothesized that a higher burden of AL would be associated with increased risk of adverse outcomes among older men with HFpEF.

## Methods

### Study participants

We recruited 4236 male veterans over the age of 60 who received physical examinations at the Chinese PLA General Hospital (Beijing, China) from March 2015 to June 2019. According to the 2021 European Society of Cardiology guidelines [19], the following criteria must be met for a diagnosis of HFpEF: patients with (1) HF symptoms and/or signs; (2) left ventricular ejection fraction (LVEF) > 50%; (3) N-terminal pro-brain natriuretic peptide (NT-proBNP) > 125 pg/mL in sinus rhythm and > 375 pg/mL in atrial fibrillation; and (4) evidence of left atrium enlargement and/or left ventricle hypertrophy or diastolic dysfunction, identified on echocardiography. Eligible patients with HFpEF were required to be in the compensatory stage without medication changes for at least 6 weeks prior to enrollment. We excluded patients with severe valvular disease, hospitalization for uncompensated HF or unstable coronary heart disease in the previous 6 weeks, heart transplantation, chronic kidney disease of stage 4 or above, severe liver disease, or those receiving palliative treatment for malignant tumors. A total of 1214 patients met the criteria for HFpEF, and we collected comprehensive baseline and follow-up data. Patients who were missing AL components, adjustment factors, or follow-up information were excluded (N = 103). The final analytical sample comprised 1111 participants. The baseline characteristics of included and excluded participants are presented in Table S1.

The present cohort study was performed with the approval of the Scientific and Ethics Review Board and was conducted in line with the ethical guidelines of the 1975 Declaration of Helsinki. Written informed consent was obtained from each patient at the time of physical examination.

### Construction of AL

On the basis of previous research [20, 21] and the availability of data in our study, we collected 12 biomarkers of the secondary outcomes of hormonal activation in response to stress and that measure the regulatory systems involved in the physiological response, so as to construct a measure of AL. The biomarkers included nutritional and metabolic markers (body mass index [BMI], fasting glucose, hemoglobin, albumin), markers of cardiovascular disease and atherosclerosis (systolic blood pressure, diastolic blood pressure, heart rate, total cholesterol, triglycerides, high‑density lipoprotein cholesterol), an inflammatory marker (neutrophil-to-lymphocyte ratio [22]), and a marker of organ dysfunction (creatinine).

We constructed the AL measure by identifying risk quartiles of the biomarkers most commonly used to study AL [23]. We defined the high-risk group as patients with the highest quartile of systolic blood pressure, diastolic blood pressure, heart rate, fasting glucose, creatinine, and neutrophil-to-lymphocyte ratio, and the lowest quartile of high-density lipoprotein cholesterol, hemoglobin and albumin. Patients with the lowest quartile of BMI, total cholesterol and triglycerides were also defined as the high-risk group because these are inversely associated with mortality in older adults [24, 25]. The cut-points of all 12 AL components are presented in Table 1.

**Table 1:** Allostatic load biomarkers and corresponding quartile cut-points

Biomarkers	Cut‑points
1) Systolic blood pressure, mm Hg	≥ 144
2) Diastolic blood pressure, mm Hg	≥ 77
3) Heart rate, beats/min	≥ 79
4) Body Mass Index, kg/m^2^	≤ 22.7
5) Fasting glucose, mmol/L	≥ 6.24
6) Total cholesterol, mmol/L	≤ 3.22
7) Triglyceride, mmol/L	≤ 0.87
8) High‑density lipoprotein cholesterol, mmol/L	≤ 1.06
9) Creatinine, µmol/L	≥ 93
10) Neutrophil-to-lymphocyte ratio	≥ 2.88
11) Hemoglobin, g/L	≤ 129
12) Albumin, g/L	≤ 42.7

Highrisk group was defined as below the 25th percentile for body mass index, total cholesterol, highdensity lipoprotein cholesterol, triglyceride, hemoglobin and albumin. Highrisk group was defined as above the 75th percentile for systolic blood pressure, diastolic blood pressure, heart rate, fasting glucose, creatinine and neutrophil-to-lymphocyte ratio

The AL score was the count of biomarkers among patients in the high-risk group, ranging from 0 (lowest) to 12 (highest). We defined three AL burden categories—low (0–2), medium (3–4), and high (5–12)—with reference to a previous study whose composition and distribution of AL were similar to those in our study [26].

### Outcomes of interest and follow-up

The primary outcome was all-cause mortality. The secondary outcomes included cardiovascular and non-cardiovascular mortality and HF admission. Cardiovascular mortality was defined as mortality owing to ischemic heart disease, congestive heart failure, stroke, malignant arrhythmia, and sudden mortality. Non-cardiovascular mortality referred to mortality from infections, cancer, other non-cardiovascular events, and unexpected mortality. For patients who were admitted multiple times owing to HF, we recorded their first admission only. As of December 31st, 2021, follow-up was performed every 6 months. Information on causes of death and hospitalization was collected through electronic medical records, and telephone interviews were conducted to avoid any missing follow-up information. Study outcomes were adjudicated by two cardiologists, and events were recorded only when both experts reached an agreement.

### Covariates

Covariates in this study included age, smoking, alcohol intake, NT-proBNP, and the number of comorbidities (atrial fibrillation, coronary heart disease, chronic kidney disease, chronic obstructive pulmonary disease, diabetes, and hypertension), which are considered potential risk factors for HFpEF [5, 27–31]; all of these were obtained from the medical records.

### Echocardiographic measurements

Echocardiographic measurements were performed using commercially available ultrasound diagnostic instruments, under the guidelines issued by the American Society of Echocardiography [32]. We performed comprehensive two-dimensional color, pulsed-wave, and continuous-wave Doppler echocardiogram. The cavity dimension and wall thickness were measured in a parasternal long-axis view. The left ventricular mass was estimated using the formula recommended in the guidelines and then normalized to the left ventricular mass index (LVMI) according to the body surface area (calculated using the formula of Stevenson). The left atrial volume was calculated using the estimated ellipsoid method [33], and then normalized to the left atrial volume index (LAVI) via the above method. LAVI > 34 mL/m^2^ and LVMI ≥ 115 g/m^2^ for men were considered evidence of left atrial enlargement and left ventricular hypertrophy, respectively. The LVEF was measured using the modified Simpson’s method in the apical four- and two-chamber views.

### Statistical analysis

We used the mean and standard deviation (SD) for continuous variables, and number and percentage for categorical variables, to describe the baseline characteristics of study participants in the three categories of AL burden (low, medium, and high). Characteristics were compared across the three groups using analysis of variance and χ^2^ tests for continuous and categorical variables, respectively. We calculated the incidence density of each outcome for each AL burden category and overall. We used Kaplan–Meier survival curves and a Cox proportional hazards model (both continuous and in categories) to determine the unadjusted and adjusted associations between AL and each outcome. We adjusted for age in the multivariable model; smoking, alcohol intake, NT-proBNP, and the number of comorbidities were additionally included in the fully adjusted model. Furthermore, subgroup analysis was conducted to examine the association between AL (continuous) and all-cause mortality among the following subgroups: age (< 80 or ≥ 80 years), obesity (BMI < 28 kg/m^2^ or ≥ 28 kg/m^2^), and comorbidities (0–1 or ≥ 2). We also examined the interactions between AL and these subgroups in the fully adjusted model. All tests were two sided with a significance level of p < 0.05. We conducted all analyses using Stata v.17.0 (StataCorp LLC, College Station, TX, USA), and GraphPad Prism 8.3.0 software (GraphPad Software Inc., San Diego, CA, USA) was used for drafting the figures.

## Results

### Baseline characteristics

A total of 1111 patients with HFpEF were included in this study. The distribution of the AL score (range: 0–12) was skewed to the right for patients (Fig. S1); only 47 (4.2%) patients had a score of 7–12. The median AL was 3 (interquartile range = 2–4) among patients. The proportion of patients with an AL score of 0–2 (low burden), 3–4 (medium burden), and 5–12 (high burden) was 43.3%, 36.1%, and 20.6%, respectively.

The mean age for patients with an AL score of 0–2, 3–4, and 5–12 was 74.7 ± 10.8, 80.0 ± 10.3, and 85.1 ± 8.1 years, respectively (p < 0.001). We observed significant differences in BMI, NT-proBNP, history of alcohol intake, proportion of statin use, left ventricular end systolic diameter, left ventricular end diastolic volume, left ventricular end systolic volume, left ventricular mass index, and the prevalence of atrial fibrillation, coronary heart disease, chronic obstructive pulmonary disease, chronic kidney disease, diabetes, and hypertension among the three groups (details are shown in Table 2).

**Table 2:** Baseline characteristics by allostatic load burden (low, medium and high)

Variable	Low (N = 481)	Medium (N = 401)	High (N = 229)	Total (N = 1111)	*p*value
Age (yrs)	74.7 ± 10.8	80.0 ± 10.3	85.1 ± 8.1	78.8 ± 10.8	< 0.001
Smoking (%)	87 (18.1)	61 (15.2)	32 (14.0)	180 (16.2)	0.300
Alcohol (%)	182 (37.8)	121 (30.2)	61 (26.6)	364 (32.8)	0.005
BMI (kg/m^2^)	25.0 ± 2.5	24.3 ± 3.3	23.6 ± 3.8	24.4 ± 3.1	< 0.001
NTproBNP (pg/mL)	429 (321–535)	430 (319–542)	445 (331–595)	433 (321–546)	0.033
*Echocardiography*					
IVS (mm)	11.1 ± 2.7	11.2 ± 2.3	11.3 ± 2.4	11.2 ± 2.5	0.310
LVPWT (mm)	10.9 ± 1.1	10.9 ± 1.0	10.7 ± 1.3	10.9 ± 1.1	0.066
LVEDD (mm)	53.2 ± 6.5	52.3 ± 5.7	52.2 ± 6.9	52.7 ± 6.3	0.051
LVESD (mm)	36.0 ± 4.5	35.2 ± 4.4	35.3 ± 4.9	35.6 ± 4.6	0.016
LAD (mm)	42.4 ± 6.5	41.4 ± 7.3	42.2 ± 7.7	42.0 ± 7.1	0.140
TR velocity (m/s)	2.8 ± 0.4	2.8 ± 0.4	2.9 ± 0.4	2.8 ± 0.4	0.500
LVEDV (mL)	139.6 ± 39.5	133.7 ± 33.8	134.1 ± 40.8	136.3 ± 37.9	0.042
LVESV (mL)	55.9 ± 17.0	52.8 ± 15.9	53.6 ± 17.8	54.3 ± 16.8	0.019
LAV (mL)	47.2 ± 15.0	45.5 ± 16.7	47.4 ± 17.9	46.7 ± 16.3	0.220
LVEF (%)	59.9 ± 5.2	60.6 ± 5.7	60.0 ± 5.5	60.2 ± 5.4	0.130
LVMI (g/m^2^)	124.4 ± 10.4	125.3 ± 10.4	126.9 ± 13.2	125.2 ± 11.1	0.018
LAVI (mL/m^2^)	25.9 ± 8.1	25.5 ± 9.3	27.2 ± 10.0	26.0 ± 9.0	0.077
RWT	0.4 ± 0.1	0.4 ± 0.1	0.4 ± 0.1	0.42 ± 0.08	0.510
*Medication (%)*					
ACEI/ARB	309 (64.2)	280 (69.8)	149 (65.1)	738 (66.4)	0.190
Beta blocker	204 (42.4)	196 (48.9)	104 (45.4)	504 (45.4)	0.160
Diuretic	199 (41.4)	204 (50.9)	112 (48.9)	515 (46.4)	0.013
Statins	329 (68.4)	286 (71.3)	150 (65.5)	765 (68.9)	0.300
*Medical history (%)*					
Atrial fibrillation	47 (9.8)	59 (14.7)	50 (21.8)	156 (14.0)	< 0.001
CHD	173 (36.0)	196 (48.9)	133 (58.1)	502 (45.2)	< 0.001
COPD	69 (14.3)	95 (23.7)	64 (27.9)	228 (20.5)	< 0.001
CKD	22 (4.6)	48 (12.0)	54 (23.6)	124 (11.2)	< 0.001
Diabetes	204 (42.4)	200 (49.9)	132 (57.6)	536 (48.2)	< 0.001
Hypertension	292 (60.7)	277 (69.1)	172 (75.1)	741 (66.7)	< 0.001
*Number of comorbidities*	1.7 ± 1.1	2.2 ± 1.2	2.6 ± 1.3	2.1 ± 1.2	
0–1 (%)	229 (47.6)	124 (30.9)	41 (17.9)	394 (35.5)	< 0.001
≥ 2 (%)	252 (52.4)	277 (69.1)	188 (82.1)	717 (64.5)	

Data are presented as mean ± SD or as percentages

BMI, body mass index; NTproBNP, Nterminal probrain natriuretic peptide; IVS, interventricular septal thickness; LVPWT, left ventricular posterior wall thickness; LVEDD, left ventricular end diastolic diameter; LVESD, left ventricular end systolic diameter; LAD, left atrial diameter; TR, tricuspid regurgitation; LVEDV, left ventricular end diastolic volume; LVESV, left ventricular end systolic volume; LAV, left atrial volume; LVEF, left ventricular ejection fraction; LVMI, left ventricular mass index; LAV, left atrial volume index; RWT, relative wall thickness; ACEI, angiotensinconverting enzyme inhibitors; ARB, angiotensin receptor antagonist; CHD, coronary heart disease; COPD, chronic obstructive pulmonary disease; CKD, chronic kidney disease

### Associations between AL and adverse outcomes

The median follow-up time was 4.6 years, during which time a total of 108 patients experienced cardiovascular mortality (N = 50) or non-cardiovascular mortality (N = 58), and 100 patients had HF admission. The overall mortality, cardiovascular mortality, and HF admission rate was 22.20 (95% confidence interval [CI]: 18.39–26.81), 10.28 (95% CI: 7.79–13.56), and 21.71 (95% CI: 17.85–26.41) per 1000 person-years, respectively. As a categorical variable, higher AL burden was associated with higher incidence of all adverse outcomes (Table 3).

**Table 3:** Association between allostatic load and Outcomes in HFpEF patients

	AL (continuous)	AL category		
		**Low**	**Medium**	**High**
*All*‑*cause mortality*				
Events per 1000 PYs (95% CI)	22.20 (18.39–26.81)	6.31 (3.74–10.66)	23.78 (17.57–32.17)	59.09 (45.03–77.55)
Unadjusted HR (95% CI)	1.53 (1.40–1.68)	Ref.	3.75 (2.05–6.88)	9.24 (5.12–16.68)
Model 1^*^: adjusted HR (95% CI)	1.37 (1.24–1.52)	Ref.	2.67 (1.45–4.91)	5.07 (2.77–9.29)
Model 2^†^: adjusted HR (95% CI)	1.31 (1.18–1.46)	Ref.	2.53 (1.37–4.68)	4.21 (2.27–7.83)
*Cardiovascular mortality*				
Events per 1000 PYs (95% CI)	10.28 (7.79–13.56)	2.71 (1.22–6.02)	12.45 (8.20–18.91)	25.00 (16.46–37.97)
Unadjusted HR (95% CI)	1.49 (1.30–1.71)	Ref.	4.58 (1.86–11.29)	9.18 (3.72–22.64)
Model 1^*^: adjusted HR (95% CI)	1.29 (1.11–1.50)	Ref.	3.02 (1.22–7.50)	4.48 (1.79–11.20)
Model 2^†^: adjusted HR (95% CI)	1.20 (1.03–1.40)	Ref.	2.67 (1.07–6.68)	3.13 (1.23–7.97)
*Non*‑*cardiovascular mortality*				
Events per 1000 PYs (95% CI)	11.92 (9.22–15.42)	3.61 (1.80–7.21)	11.32 (7.30–17.55)	34.09 (23.84–48.76)
Unadjusted HR (95% CI)	1.57 (1.39–1.79)	Ref.	3.13 (1.38–7.12)	9.28 (4.25–20.26)
Model 1^*^: adjusted HR (95% CI)	1.45 (1.26–1.66)	Ref.	2.39 (1.04–5.49)	5.74 (2.56–12.90)
Model 2^†^: adjusted HR (95% CI)	1.46 (1.26–1.69)	Ref.	2.45 (1.06–5.63)	5.81 (2.55–10.28)
*Heart failure admission*				
Events per 1000 PYs (95% CI)	21.71 (17.85–26.41)	5.95 (3.45–10.24)	27.92 (20.91–37.28)	53.07 (39.08–72.08)
Unadjusted HR (95% CI)	1.50 (1.36–1.65)	Ref.	4.58 (2.48–8.49)	8.46 (4.53–15.80)
Model 1^*^: adjusted HR (95% CI)	1.32 (1.19–1.47)	Ref.	3.22 (1.73–5.98)	4.49 (2.38–8.47)
Model 2^†^: adjusted HR (95% CI)	1.24 (1.11–1.39)	Ref.	2.68 (1.43–5.01)	3.24 (1.69–6.23)

^*^ Model 1 adjusted by age;

^†^ Model 2 include age, smoking, alcohol, NT‑proBNP and the number of comorbidities

AL, allostatic load; PY, person‑year; HR, hazard ratio; CI, confidence interval

In the fully adjusted Cox proportional hazards model, the per-unit higher AL score was significantly associated with a 31%, 20%, 46%, and 24% higher risk of all-cause mortality, cardiovascular mortality, non-cardiovascular mortality, and HF admission among patients, respectively (Table 3).

**Fig. 1:**
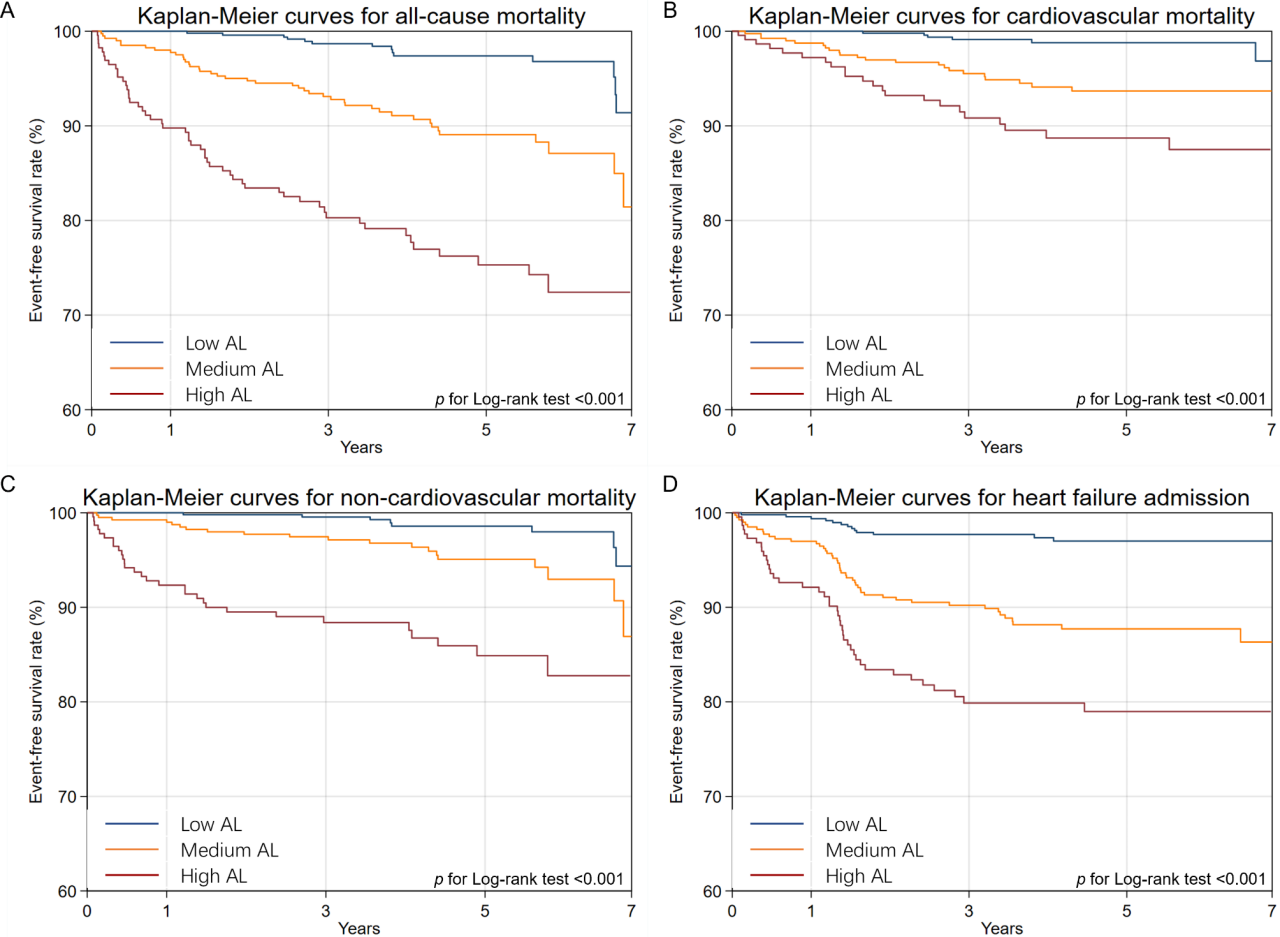
Kaplan‑Meier survival curves for each outcome in heart failure with preserved heart failure patients with different allostatic load categories. Event‑free survival rates from (**A**) all‑cause mortality, (**B**) cardiovascular mortality, (**C**) non‑cardiovascular mortality, and (**D**) heart failure admission

Kaplan–Meier survival curves for each predefined outcome in patients with HFpEF according to AL category are shown in Fig. 1. The unadjusted risks of all-cause mortality (Fig. 1A), cardiovascular mortality (Fig. 1B), non-cardiovascular mortality (Fig. 1C), and HF admission (Fig. 1D) differed significantly among the different AL categories. In the fully adjusted model, patients with a medium AL burden had a 2.53 (95% CI: 1.37–4.68), 2.67 (95% CI: 1.07–6.68), 2.45 (95% CI: 1.06–5.63), and 2.68 (95% CI: 1.43–5.01) times greater risk of all-cause mortality, cardiovascular mortality, non-cardiovascular mortality, and HF admission, respectively, compared with patients who had a low AL burden. Patients with a high AL burden had a 4.21 (95% CI: 2.27–7.83), 3.31 (95% CI: 1.23–7.97), 5.81 (95% CI: 2.55–10.28), and 3.24 (95% CI: 1.69–6.23) times greater risk of each of the above outcomes, respectively, compared with patients who had a low AL burden.

### Subgroup analysis

**Fig. 2:**
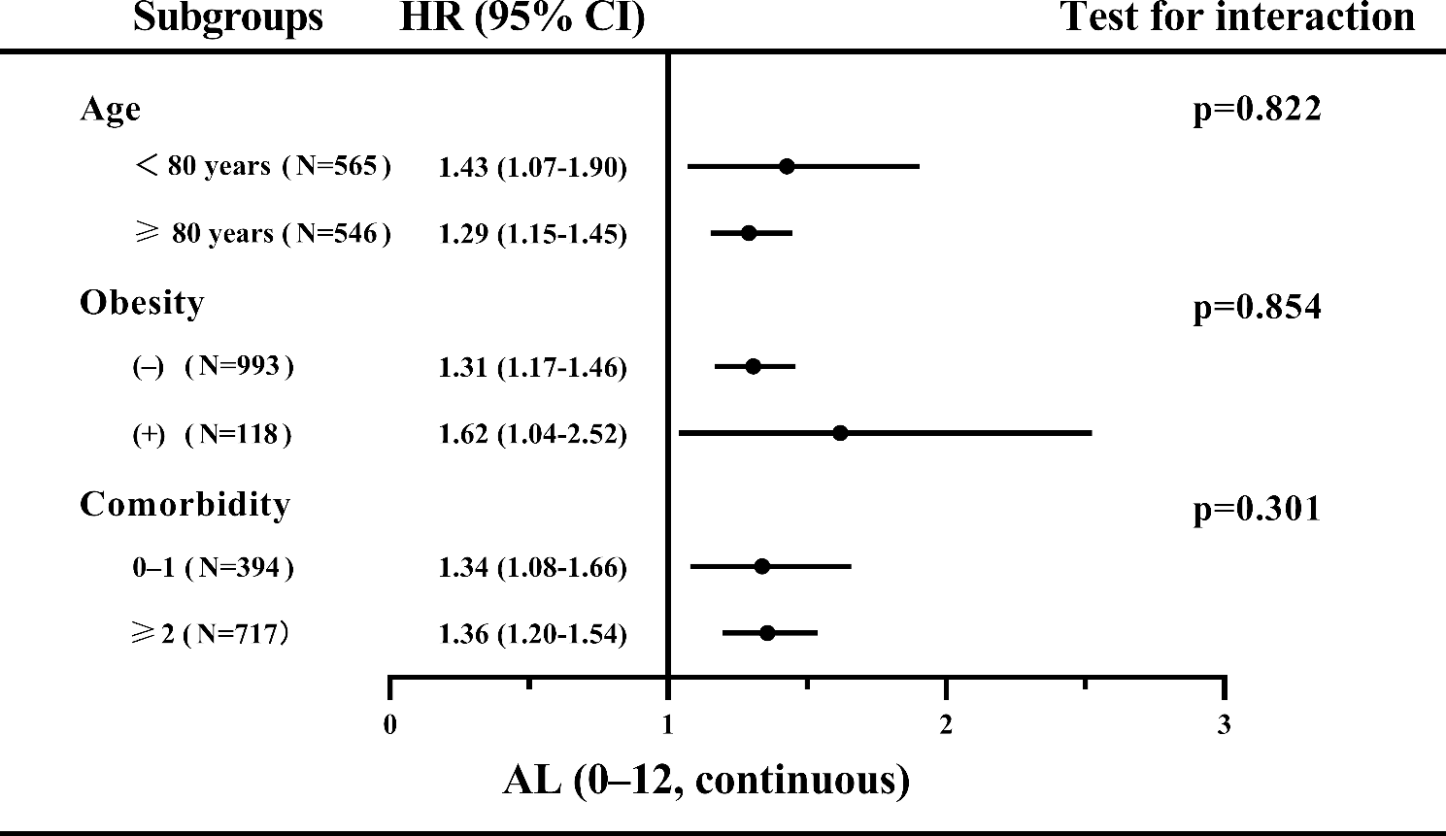
Association between allostatic load and all‑cause mortality in the subgroups. AL, allostatic load; HR, hazard ratio; CI, confidence interval

We also examined the association between AL (continuous) and all-cause mortality in different subgroups, as shown in Fig. 2. In the fully adjusted model, AL remained an independent risk factor for all-cause mortality in each subgroup. Additionally, no significant interactions were observed between AL and age, obesity, or comorbidity subgroups (Fig. 2).

## Discussion

In the present study, we aimed to explore the association between AL and adverse outcomes in older Chinese men with HFpEF. Our results indicated that AL was an independent risk factor for all-cause mortality, cardiovascular mortality, non-cardiovascular mortality, and HF admission in these patients.

These finding were somewhat consistent with those of previous evidence. AL is reported to be a strong contributor to premature death in the United States [16]. A higher AL score has been found to significantly increase the 10-year mortality risk among older adults in Taiwan, regardless of the cause of death [34]. Most previous studies on AL have used public data from large-sample health surveys [10, 11, 16, 34]. Although these studies have incomparable advantages owing to their large sample size and representativeness, the role of AL in specific populations is often overlooked. To our knowledge, only a few studies have focused on the role of AL in specific populations with cardiovascular disease. Previous studies have reported that patients with essential hypertension and coronary heart disease who have allostatic overload have a higher disease-related emotional burden, higher prevalence of psychosis, and poorer psychosocial functioning [35]. Similarly, allostatic overload is associated with increased psychological distress in patients with atrial fibrillation [36]. Allostatic overload at baseline was found to be an independent risk factor for complications and mortality after implantation in patients with an implantable cardioverter defibrillator [37]. However, in the above study, semi-structured interviews were used to define allostatic overload rather than biomarkers [38]. Other studies have shown that AL overload has a negative effect in patients with essential hypertension, whether defined using interviews [39] or biomarkers [40]. Our study was the first to explore the association between AL and adverse outcomes in patients with HFpEF, adding to the evidence that AL can predict adverse outcomes not only in the general population but also in patients with specific diseases.

The results of our subgroup analysis suggested that the association between AL and adverse outcomes remained robust regardless of stratification by age, BMI, or number of comorbidities. The results of an interaction test also suggested that the predictive effect of AL on adverse outcomes was not affected by age, obesity, or comorbidity. Notably, in this study, we defined the high-risk biomarker group using the quartile risk method, which reflects physiological disorders, rather than using clinical cutoff values used to diagnose disease. Therefore, high AL cannot simply be interpreted as a greater comorbidity burden.

Interestingly, AL in patients with HFpEF was not limited to predicting cardiovascular-related adverse events but it could also predict non-cardiovascular mortality. This may be attributed to the observation that among patients who died of non-cardiovascular causes in this study, the majority of specific causes of death were pneumonia and cancer. Most of the biomarkers utilized to construct the AL in this study were related to the prognosis of these two diseases. In addition, previous studies have demonstrated the correlation between AL and mortality associated with both pneumonia and cancer [34, 41]. Thus, AL may be a useful prognostic tool not only for cardiovascular mortality but also for non-cardiovascular mortality in older male patients with HFpEF.

The AL relies on information that is easily available from physical examinations and laboratory parameters, making it a practical approach for risk stratification of patients with HFpEF in various healthcare and clinical settings.

A strength of this study is that it was the first to explore the association between AL and adverse outcomes in patients with HFpEF, further complementing the role of AL in populations with specific cardiovascular diseases. Our cohort was generally older, with approximately half of patients over 80 years old, which is another strength compared with previous studies on AL. Furthermore, there was only minor loss to follow-up in this study, which supports the reliability of our conclusions.

This study had several limitations. First, this was a single-center study conducted among older male patients. Whether the conclusions in our study can be applied to women, younger patients or other populations must be further confirmed in future studies. Second, this study may have underestimated the prevalence of HFpEF since echocardiographic measures of diastolic dysfunction were not available. Third, the determination of AL lacks a gold standard, and the quantity and type of AL components varied across previous studies. The biomarkers used to construct the measure of AL in this study were all obtained in regular physical examinations, which increases their feasibility in clinical and nursing practice. However, owing to the lack of neuroendocrine markers originally used to construct the AL measure, caution is needed in directly comparing the results of this study with those of other studies. Additionally, dynamic changes in the AL components were not assessed in this study; future longitudinal studies are needed to further determine the impact of dynamic changes in AL on the prognosis of patients with HFpEF.

## Conclusions

Our study findings showed that a higher burden of AL was associated with an increased risk of poor prognosis in older male patients with HFpEF. AL relies on information that is easily obtained in regular physical examinations and it can be assessed in various care and clinical settings to help risk stratification of HFpEF patients.

## Electronic supplementary material

Below is the link to the electronic supplementary material.

Supplementary Material 1

## Data Availability

The datasets used and/or analyzed during the current study are available from the corresponding author on reasonable request.

## Abbreviations

AL: allostatic load
BMI: body mass index
HF: heart failure
HFpEF: heart failure with preserved ejection fraction
LAVI: left atrial volume index
LVEF: left ventricular ejection fraction
LVMI: left ventricular mass index
NT-proBNP: N-terminal pro-brain natriuretic peptide
SD: standard deviation.
