# Supplementary material for: Association between allostatic load and adverse outcomes among older patients with heart failure with preserved ejection fraction
Source: BMC Geriatr. 2023 Jun 9;23:360. doi: 10.1186/s12877-023-04091-x (PMC10257257; doi:10.1186/s12877-023-04091-x)
Supplement: Supplementary file 1 — Supplementary Material 1 [file 12877_2023_4091_MOESM1_ESM.docx]

**Association between** **allostatic load and adverse outcomes among older patients with heart failure with preserved ejection fraction**

1. **Fig.S1** Distribution of sample size by allostatic load (AL) score among HFpEF patients.
2. **Table S1** Baseline characteristics between included and excluded participants


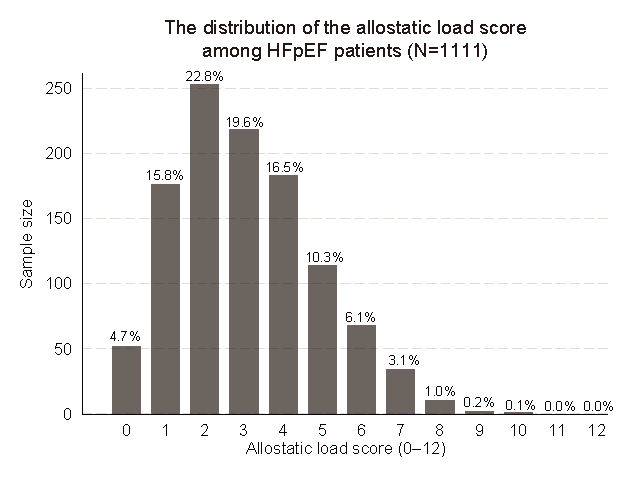


**Fig.S1** Distribution of sample size by allostatic load (AL) score among HFpEF patients. The distribution of the AL score (range: 0–12) is right-skewed for HFpEF patients; only 47 (4.2%) HFpEF patients had a score of 7–12.

**Table S1** Baseline characteristics between included and excluded participants

| Variable | Included  (N=1111) | Excluded  (N=103) | *p*‑value |
| --- | --- | --- | --- |
| Age (yrs) | 78.8±10.8 | 80.8±10.4 | 0.070 |
| Smoking (%) | 180 (16.2) | 13 (12.7) | 0.342 |
| Alcohol (%) | 364 (32.8) | 28 (27.2) | 0.247 |
| BMI (kg/m^2^) | 24.4±3.1 | 25.3±3.2 | 0.011 |
| NT‑proBNP (pg/mL) | 433 (321–546) | 414 (272–542) | 0.276 |
| *Echocardiography* |  |  |  |
| IVS (mm) | 11.2±2.5 | 11.6±2.2 | 0.105 |
| LVPWT (mm) | 10.9±1.1 | 10.7±1.0 | 0.137 |
| LVEDD (mm) | 52.7±6.3 | 52.6±5.7 | 0.824 |
| LVESD (mm) | 35.6±4.6 | 35.0±4.0 | 0.207 |
| LAD (mm) | 42.0±7.1 | 41.1±7.3 | 0.212 |
| TR velocity (m/s) | 2.8±0.4 | 2.8±0.4 | 0.698 |
| LVEDV (mL) | 136.3±37.9 | 134.9±33.3 | 0.717 |
| LVESV (mL) | 54.3±16.8 | 51.9±13.9 | 0.154 |
| LAV (mL) | 46.7±16.3 | 44.7±16.9 | 0.257 |
| LVEF (%) | 60.2±5.4 | 61.6±4.0 | 0.009 |
| LVMI (g/m^2^) | 125.2±11.1 | 126.6±11.8 | 0.230 |
| LAVI (mL/m^2^) | 26.0±9.0 | 24.7±8.8 | 0.153 |
| RWT | 0.42±0.08 | 0.41±0.07 | 0.389 |
| *Medication (%)* |  |  |  |
| ACEI/ARB | 738 (66.4) | 63 (61.2) | 0.281 |
| Beta blocker | 504 (45.4) | 41 (39.8) | 0.278 |
| Diuretic | 515 (46.4) | 48 (46.6) | 0.962 |
| Statins | 765 (68.9) | 60 (58.2) | 0.027 |
| *Medical history (%)* |  |  |  |
| Atrial fibrillation | 156 (14.0) | 21 (20.4) | 0.081 |
| CHD | 502 (45.2) | 40 (38.8) | 0.215 |
| COPD | 228 (20.5) | 20 (19.4) | 0.790 |
| CKD | 124 (11.2) | 11 (10.7) | 0.882 |
| Diabetes | 536 (48.2) | 54 (52.4) | 0.417 |
| Hypertension | 741 (66.7) | 78 (75.7) | 0.061 |
| *Number of comorbidities* | 2.1±1.2 | 2.2±1.1 | 0.354 |
| 0–1 (%) | 394 (35.5) | 34 (33.0) | 0.618 |
| ≥2 (%) | 717 (64.5) | 69 (67.0) | 0.618 |

Data are presented as mean ± SD or as percentages.

BMI, body mass index; NT‑proBNP, N‑terminal pro‑brain natriuretic peptide; IVS, interventricular septal thickness; LVPWT, left ventricular posterior wall thickness; LVEDD, left ventricular end diastolic diameter; LVESD, left ventricular end systolic diameter; LAD, left atrial diameter; TR, tricuspid regurgitation; LVEDV, left ventricular end diastolic volume; LVESV, left ventricular end systolic volume; LAV, left atrial volume; LVEF, left ventricular ejection fraction; LVMI, left ventricular mass index; LAV, left atrial volume index; RWT, relative wall thickness; ACEI, angiotensin-converting enzyme inhibitors; ARB, angiotensin receptor antagonist; CHD, coronary heart disease; COPD, chronic obstructive pulmonary disease; CKD, chronic kidney disease.
